# Ethylene Oxide Measurements From OSHA Workplace Investigations: Patterns in Exposure by Industry, Occupation, and Over Time

**DOI:** 10.1002/ajim.70008

**Published:** 2025-07-21

**Authors:** Brian Christensen, Matthew M. Dahm, I‐Chen Chen, Kaitlin Kelly‐Reif

**Affiliations:** ^1^ National Institute for Occupational Health and Health (NIOSH) Cincinnati Ohio USA

**Keywords:** ethylene oxide, OSHA, sterilization, surveillance, workplace exposure

## Abstract

**Background:**

Ethylene oxide (EtO) is an occupational carcinogen; however, contemporary exposure scenarios in US workplaces are not well characterized within the literature. We aim to describe EtO exposure trends in the US workplace using historical data from the Occupational Safety and Health Administration (OSHA) Chemical Exposure Health Database and the OSHA Information System.

**Method:**

We collated and analyzed air sampling data collected between 1979 and 2020 to characterize exposures across key industries and occupations. We evaluated exposure by industry and occupational category, examined changes in exposures over time, and quantified the proportion of samples that exceeded existing occupational exposure limits.

**Results:**

Exposures generally decreased over time. However, the magnitude and pattern of reductions were not consistent across industries. Within the aggregated dataset, approximately 46% of samples exceeded the National Institute for Occupational Safety and Health recommended exposure limit (NIOSH REL) of 0.1 parts per million (ppm), and approximately 18% of samples exceeded the OSHA permissible exposure limit (PEL) of 1 ppm. 70% of samples collected from industrial sterilization workplaces exceeded the NIOSH REL. Exposure data was limited between 2000 and 2020, most notably for the health services and chemical manufacturing industries.

**Conclusions:**

Contemporary EtO exposures for key industries and occupational categories are poorly characterized. Still, exposures in the industrial sterilization industry appear higher than in other industries and have proportionally declined the least over time. Additional exposure assessment research and further efforts in occupational risk assessment are important to better understand the health burdens of workers exposed to EtO.

## 1 Introduction

Ethylene oxide (EtO) is produced for an array of industrial and medical applications. In the United States, EtO is predominantly used as an intermediate in the production of ethylene glycols, but a substantial proportion of EtO is used to produce surface‐active agents such as ethoxylates, glycol ethers, ethanolamines, and polyether polyols [1, 2]. These surface‐active agents are used in the production of detergents, surfactants, emulsifiers, and dispersants. A smaller proportion of synthesized EtO is used to sterilize medical equipment or fumigate agricultural products (< 0.05%) [2, 3]. Nevertheless, separate reports from the Food and Drug Administration (FDA) and the Occupational Safety and Health Administration (OSHA) estimate that approximately 50% (~20 billion devices) of all medical equipment are sterilized and 15%–22% of all spices are fumigated using EtO [4, 5].

Commercial EtO production began in 1914, and the scale of production has steadily increased over time, both nationally and globally [1]. A report cited in a review of the EtO literature by the Agency for Toxic Substances and Disease Registry reported that the United States produced approximately 5–10 billion pounds of EtO in 2015 [6]. As of 2019, the Environmental Protection Agency's (EPA) Toxic Reports Inventory (TRI) documented over 100 facilities that produce, process, or use EtO in the United States. Approximately 15 of those facilities directly manufacture EtO [5, 6].

EtO exposures are well‐documented in the chemical manufacturing, health services, and industrial sterilization industries [6]. Workers who handle sterilized equipment or are directly involved in medical equipment sterilization account for most of the EtO‐exposed workforce [7]. While registered nurses represent the largest proportion of health service workers [7], only a small proportion of nursing tasks involve handling sterilized medical instruments. Nursing assistants, medical assistants, surgical technologists, and surgical assistants are more likely to be in frequent contact with sterilized instruments than nurses, but exposures associated with handling sterilized instruments have generally approximated background exposure [8]. Sterilization operators working in the central processing unit of health service facilities make up a smaller proportion of health service workers but have the highest potential for elevated EtO exposure. Working zone air sampling results for sterilization operators within health service facilities have frequently exceeded 1 ppm historically [9]. Workplace tasks that contribute to sterilization operators' heightened exposure potential include loading and unloading of medical equipment into sterilizers and the transfer of sterilized equipment [10, 11, 12]. While industrial sterilization workers make up a smaller proportion of the US workforce than health service workers, the potential for elevated occupational exposures among industrial sterilization workers is thought to be higher due to the increased scale (volume) and frequency of sterilizing operations.

**Figure 1:**
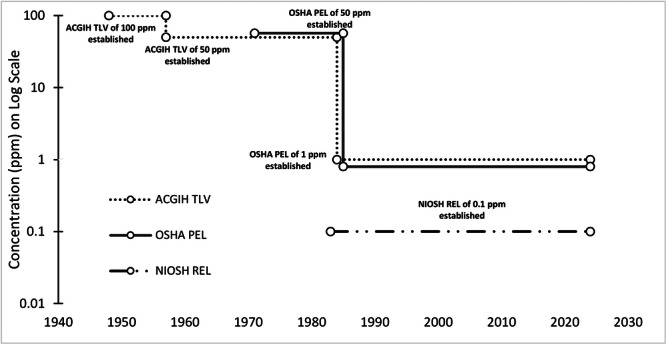
Timeline of when US occupational exposure limits were established. ACGIH TLV: American Conference of Governmental Industrial Hygienists threshold limit value; OSHA PEL: Occupational Safety and Health Administration permissible exposure limit; NIOSH REL: National Institute for Occupational Safety and Health recommended exposure limit; ppm: parts per million.

Early concerns about workplace EtO exposure focused on acute health effects such as respiratory irritation and neurotoxicity [13]. These findings prompted the American Conference of Governmental Industrial Hygienists (ACGIH) to establish an 8‐h time‐weighted average (TWA) threshold limit value (TLV) of 50 parts per million (ppm) in 1957. Shortly after its establishment, OSHA adopted the ACGIH TLV of 50 ppm (1971) [14]. A large body of animal and occupational epidemiological research in the 1970s and 1980s led to the determination that EtO is a human carcinogen [15]. Evidence of EtO carcinogenicity led to the development of a National Institute for Occupational Safety and Health recommended exposure limit (NIOSH REL) of 0.1 ppm in 1983 and an updated OSHA PEL of 1 ppm in 1984 [16, 17, 18, 19, 20, 21]. The NIOSH REL remains the most protective occupational exposure guideline in the United States and globally (See Table S1 for the United States and international OELs) [22, 23, 24, 25]. NIOSH and OSHA established short‐term exposure limits for EtO at 5 ppm in 1983 and 1988, respectively (10 min for NIOSH ceiling limit and 15 min for OSHA excursion limit) [25, 26]. The chronology of when full‐shift US OELs were established is illustrated in Figure 1.

Historically, several exposure assessment studies of health service sterilization workers, industrial sterilization workers, and chemical manufacturing workers reported EtO exposures in exceedance of the current OSHA PEL (1 ppm), frequently by multiple orders of magnitude [10, 21, 27, 28, 29, 30]. Studies reporting OSHA PEL exceedance with high frequency were largely published between the 1970s and 1990s and predominantly conducted outside of the United States. Given the lack of EtO air monitoring data published within the last 30 years, findings from studies conducted between the 1970s and 1990s largely constitute our quantitative understanding of EtO exposure potential in the workplace today. However, it is unclear whether exposures have decreased across relevant industries and occupations, and if so, to what extent. We aim to fill some of these gaps in knowledge through a comprehensive analysis of three OSHA air monitoring datasets. Findings from our analyses will (1) improve our understanding of occupational EtO exposure by industry, (2) identify relevant workers with the highest EtO exposure potential, and (3) evaluate temporal trends in occupational EtO exposure. Additionally, we will identify remaining knowledge gaps in occupational EtO exposure characterization.

## 2 Methods

### 2.1 OSHA Compliance and State Consultation Data

We analyzed OSHA's Chemical Exposure Health (CEH) database and the OSHA Information System (OIS), known as the Integrated Management Information System (IMIS) before 2014. The IMIS/OIS includes an enforcement dataset and a dataset from OSHA's state consultation program. This activity was reviewed by the CDC, deemed not research, and was conducted in accordance with applicable federal law and CDC policy^§^ ^1^. To the best of our knowledge, the combination of the OSHA CEH and IMIS/OIS databases^2^ is the most comprehensive set of existing occupational EtO air monitoring data for historical and contemporary US workplaces. The datasets were combined into one aggregate dataset by matching inspection and sampling numbers.

The OSHA CEH dataset consists of data collected during OSHA compliance visits, whereas the IMIS/OIS dataset consists of data collected during OSHA compliance visits and data collected at the request of employers seeking assistance in characterizing EtO exposures in their workplaces [31, 32]. The state consultation data and enforcement data within the IMIS/OIS are delineated into two separate datasets. There was significant overlap (determined by matching inspection and sampling numbers) between the OSHA CEH dataset and the IMIS/OIS compliance dataset. IMIS/OIS results that were associated with the same inspection and sample numbers as OSHA CEH results were excluded from analysis. Each dataset included inspection number, industry classification (North American Industry Classification System [NAICS] or Standard Industry Classification [SIC]), date of sample collection, sampling type (personal air, area air, urine, or bulk), and data qualifiers (e.g., blank, non‐detect, and backup sample). Additionally, company name, sample duration, flow rate, sampling method, sample duration, job title, citation status, and type of visit were reported in at least one of the datasets, but not all three.

Industry classification codes were aggregated into one of three categories: health service, chemical production, and industrial sterilization, using SIC codes. NAICS codes were converted into SIC codes using the NAICS/SIC crosswalks [33]. See Tables S2a–S2c within the Supporting Information for how SIC codes were aggregated into categories. Job titles were aggregated into seven similar exposure groups (SEGs) by industry: *chemical operators* representing the chemical manufacturing industry; *animal service workers, health services central processing workers*, and *health services noncentral processing workers* representing the health services industry; and *industrial sterilization operators, industrial sterilization production floor workers, and industrial sterilization warehousing workers* representing the industrial sterilization industry (see Table S3 within the Supporting Information for how SEGs were defined). Because area sampling results only accounted for ~20% of the aggregated dataset and median results were similar across sampling type (see Table S5 for results by sampling type), PBZ and area sample results were evaluated together for analyses of the entire aggregate dataset and analyses by industry and over time. Data points that did not contain job title information and area samples were excluded from analyses of occupational categories.

Bulk, blank, urine, invalid, undocumented, unanalyzed, and backup samples were removed from the dataset to improve the applicability of the sample results. Passive samples represented a small proportion of the CEH dataset (~6%). Moreover, due to method limitations (OSHA method no longer used or supported by OSHA personnel) and data analysis limitations (cannot censor values below LOQ using sample volume), passive samples within the CEH dataset were removed. Because sample volume and sample flow rate were not provided in the IMIS/OIS datasets, passive samples could not be removed from the IMIS/OIS state consultation or enforcement datasets. Additionally, samples collected outside of the three key industries were not analyzed. A breakdown of the inclusion and exclusion criteria is included in Figure S1 within the Supporting Information.

### 2.2 Statistical Analyses

Using histograms, we determined that each dataset individually, and in aggregate, approximated a bimodal distribution pattern because of the high proportion of non‐detects, but a log‐normal distribution pattern for the second peak because of the high proportion of outlier values present in each dataset (see Figure S2 for histograms). For the CEH dataset, the limit of detection (LOD) and limit of quantitation (LOQ) for each sample were calculated based on the sample volume collected and sensitivity of the OSHA method used at the time of collection (see Table S4 for OSHA methods used to calculate LOD values for each sample). The LODs for the CEH dataset were weighted differently based on the number of samples. Given that sample duration and volume were not provided within the IMIS/OIS datasets, the OSHA method LOD at the time of collection was applied for imputation. Each censored or non‐detectable value was imputed using the multiple random value imputation method [34].

After the non‐detectable values were imputed, we calculated descriptive statistics, including measures of central tendency, spread, detection frequency, and exceedance fractions of US OELs for the aggregate dataset, industry, occupational category, and time period of collection (Table 1). Summary statistics of the individual datasets, sample type (area vs. personal air sample), citation status, type of visit (initial vs. follow‐up), and sample duration (minutes) are provided in Table S5. Differences were depicted visually using box plots and scatter plots. Marginal median regression models accounting for the statistical correlation among repeated measurements from the same company were performed to measure differences in median EtO concentrations across industries, occupations, and various time periods [35]. These values were calculated to explore whether overall and temporal differences in workplace EtO concentrations captured during compliance inspections or state consultation visits were present and to what extent. All descriptive statistics, figures, and inferential analyses were performed in Microsoft Excel, SAS version 9.4 (SAS Institute, Cary, North Carolina, the United States), or R version 4.4.0, using the *ggplot2* and *marlod* packages [36, 37]. Statistical tests were two‐sided at the 0.05 significance level.

**Table 1:** Airborne ethylene oxide concentrations stratified by time and occupational characteristics.

Category	# of measurements	Relative % of Samples	DF%	Median (ppm)	*p* value*	GM (ppm)	GSD	% > PEL	% > REL
Entire dataset (1979–2020)	2182	100%	61%	0.0600	—	0.0151	97.2	18%	46%
1979–1999	1739	80%	62%	0.0587	Reference	0.0148	97.8	18%	45%
2000–2020	443	20%	59%	0.0840	0.1423	0.0173	93.1	16%	51%
Comparison by decade									
1979–1989	1252	57%	62%	0.0554	Reference	0.0138	99.8	18%	45%
1990–1999	659	30%	59%	0.0586	0.9841	0.0167	94.2	19%	45%
2000–2009	173	8%	70%	0.1949	0.1103	0.0369	79.9	19%	56%
2010–2020	98	5%	50%	0.0269	0.9616	0.0053	97.8	12%	42%
Comparison by industry (1979–2020)									
Industrial sterilization	669	31%	81%	0.4061	Reference	0.1342	52.3	33%	70%
Chemical manufacturing	239	11%	48%	0.0050	**< 0.0001**	0.0042	130.9	14%	38%
Health services	1274	58%	53%	0.0199	**< 0.0001**	0.0061	83.0	11%	35%
Similar exposure groups by industry^†^									
Chemical manufacturing workers	203	12%	54%	0.0186	—	0.0052	143.3	15%	43%
Animal service workers	45	3%	51%	0.0100	—	0.0048	158.0	16%	40%
Health services central processing workers	508	29%	51%	0.0171	Reference	0.0037	102.9	10%	34%
Health services noncentral processing workers	181	10%	45%	0.0008	**0.0318**	0.0016	66.0	7%	23%
Industrial sterilization operators	171	10%	84%	0.7200	Reference	0.2824	62.3	43%	79%
Industrial sterilization floor production workers	103	6%	79%	0.1800	**0.0031**	0.0426	2.2	21%	59%
Industrial sterilization warehousing workers	74	4%	84%	0.2362	**0.0031**	0.0790	54.6	20%	68%
Undefined workers	457	26%	63%	0.1004	—	0.0459	34.9	21%	63%

*Note:* Statistical significance is defined by *p* values < 0.05 are bolded. Denominator for similar exposure group is 1742; Median of 1990–1999 was not significantly different than median of 2000–2009 (*p* value = 0.1195) or median of 2010–2020 (*p* value = 0.9593); Medians of 2000–2009 and 2010–2020 were not significantly different (*p* value = 0.3279). Median levels for IS floor production workers and IS warehousing workers were not significantly different (*p* value = 0.2279).

Abbreviations: % > PEL: percentage of samples that exceeded the OSHA permissible exposure limit; % > REL: percentage of samples that exceed the NIOSH recommended exposure limit; DF%: EtO detection frequency as a percentage; GM: geometric mean; GSD: geometric standard deviation; ppm: parts per million; Undefined workers: samples represent health services, industrial sterilization, and chemical manufacturing industries, but occupation was not reported.

*Marginal median regression model with a compound symmetry working correlation structure was performed for the tests of significance.

## 3 Results

### 3.1 Overall Summary Findings

We analyzed 2182 EtO air sample results representing 734 unique OSHA investigations between 1979 and 2020 (combination of OSHA inspections and state consultation visits). Descriptive statistics of the OSHA EtO dataset stratified by industry, decade of collection, and SEG are shown in Table 1. Most samples were collected at health service facilities (58.4%), followed by industrial sterilization settings (30.6%) and chemical manufacturing facilities (11.0%), but the relative proportion of collected samples for individual industries varied across time periods. Moreover, between 2000 and 2020, the industrial sterilization accounted for the largest proportion of collected samples (62%), followed by the health service industry (23%) and the chemical manufacturing industry (15%).

Health service workers operating in the central processing area accounted for 23% of all air samples, the largest proportion amongst occupational categories, followed by chemical manufacturing workers (9%), health service noncentral processing workers (8%), industrial sterilization operators (8%), industrial sterilization floor production workers (5%), industrial sterilization warehousing workers (3%), and animal service workers (2%). Area samples accounted for an additional 20% of air sample results, and undefined occupations accounted for the remaining 21% of air sample results.

For the entire dataset, the detection frequency was 61% and the exceedance fractions for the OSHA PEL and NIOSH REL were 18% and 46%, respectively. Both the detection frequency and OEL exceedance fractions were highest in the industrial sterilization industry, irrespective of the time period of collection (Table 1).

**Figure 2:**
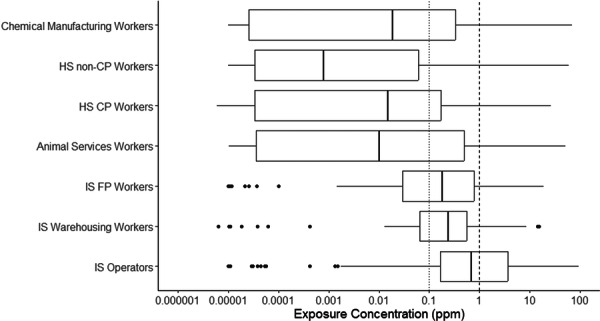
Box plot of ethylene oxide exposure by occupational category. CP, central processing; FP, floor production; HS, health services; IS, industrial sterilization; ppm, parts per million. 0.1 ppm dotted line represents National Institute for Occupational Safety and Health recommended exposure limit (NIOSH REL); 1.0 ppm dashed line represents Occupational Safety and Health Administration permissible exposure limit (OSHA PEL).

For both health service and industrial sterilization settings, workers involved directly in sterilization‐related activities were associated with elevated median concentrations in comparison to workers not involved directly in sterilization‐related activities (see Table 1). By comparison, OSHA PEL and NIOSH REL exceedance fractions were also elevated for workers involved in sterilization. As illustrated in Table 1 and Figure 2, differences in median concentrations were most pronounced across health service SEGs. Workers operating in the central processing area exhibited a median concentration that was two orders of magnitude higher than workers operating outside of the central processing area (*p* value = 0.0318). Even so, industrial sterilization operators exhibited a median exposure concentration more than three times higher than the warehousing workers and four times higher than the floor production workers (both *p* values = 0.0031). Chemical manufacturing workers exhibited higher median exposure concentrations than health service workers, but their exposures were still an order of magnitude below those reported for industrial sterilization workers, irrespective of SEGs for the industrial sterilization industry.

### 3.2 Temporal Findings

**Figure 3:**
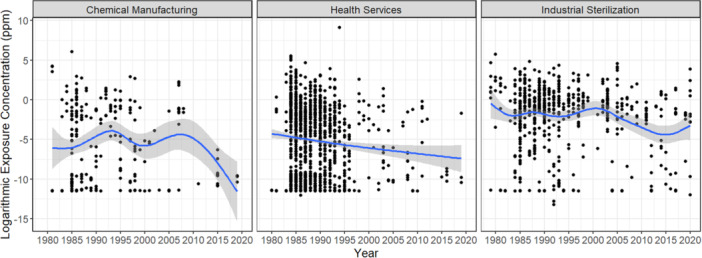
Log ethylene oxide results over time by industry. Each blue line represents a local regression (loess) trend line fitted to the data representing each industry. The gray banding around each blue line represents the confidence intervals for fitted loess trend lines.

**Figure 4:**
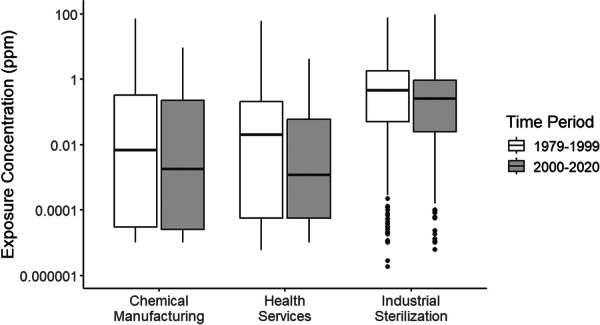
Box plot of ethylene oxide exposure by industry before and after 2000. ppm: parts per million.

For the entire dataset, EtO concentrations appear to have increased over time (see Table 1). However, this increase was confounded by the higher proportion of samples collected in industrial sterilization settings between 2000 and 2020 (62.4%). Exposures decreased over time for individual industries (see Table 2 and Figure 2). Nevertheless, the onset and extent of reductions varied across industries (see Table 2). As illustrated in Figure 3, we observed reductions within the health service industry from 1979 to 2020, with sharp reductions visible in the 1980s and 1990s. Whereas EtO exposure concentrations within the chemical manufacturing and industrial sterilization industries peaked between the late 1990s and mid‐2000s before decreasing, albeit non‐monotonically, thereafter (see Figure 3). Across individual industries, significant reductions in median EtO concentrations between 1979–1999 and 2000–2020 were only captured for the chemical manufacturing industry (*p* value: 0.0019). However, visible (Figure 4) and measurable (Table 2), but not significant, reductions were captured for the industrial sterilization (*p* value: 0.1901) and health service (*p* value: 0.3457) industries (see Table 1).

**Table 2:** Airborne ethylene oxide concentrations stratified by industry and time.

Category	# of measurements	Relative % of samples	DF%	Median (ppm)	*p* value	GM (ppm)	GSD	% > PEL	% > REL
Chemical manufacturing	239								
1979–1989	118	49%	42%	0.0009	0.2575	0.0026	179.8	15%	36%
1990–1999	80	33%	55%	0.0632	0.9515	0.0122	83.7	15%	45%
2000–2009	27	11%	78%	0.0447	Reference	0.0127	93.2	11%	48%
2010–2020	14	7%	0%	NA	—	NA	0.0	0%	0%
1979–1999	198	83%	47%	0.0083	Reference	0.0048	140.5	15%	39%
2000–2020	41	17%	51%	0.0018	**0.0019**	0.0021	90.7	7%	32%
Heath services	1274								
1979–1989	857	67%	57%	0.0300	0.8801	0.0073	87.3	13%	38%
1990–1999	356	28%	46%	0.0115	0.6867	0.0049	78.8	8%	29%
2000–2009	45	4%	33%	0.0012	0.6086	0.0019	40.2	4%	18%
2010–2020	16	1%	38%	0.0001	Reference	0.0019	65.4	0%	25%
1979–1999	1213	90%	54%	0.0200	Reference	0.0064	84.9	12%	36%
2000–2020	61	10%	34%	0.0012	0.3457	0.0018	44.4	20%	33%
Industrial sterilization	669								
1979–1989	277	42%	84%	0.4360	**0.0179**	0.2070	35.9	36%	70%
1990–1999	223	33%	81%	0.5300	**0.0216**	0.1360	60.9	36%	71%
2000–2009	101	15%	84%	0.4530	**0.0339**	0.1560	50.2	27%	75%
2010–2020	68	10%	63%	0.1230	Reference	0.0180	90.8	18%	54%
1979–1999	500	75%	83%	0.4652	Reference	0.1716	45.9	36%	71%
2000–2020	169	25%	76%	0.2498	0.1901	0.0649	72.9	23%	67%

*Note:* Statistical significance is defined by *p* values < 0.05 are bolded.

Abbreviations: % > PEL: percentage of samples that exceeded the OSHA permissible exposure limit; % > REL: percentage of samples that exceeded the NIOSH recommended exposure limit; DF%: EtO detection frequency as a percentage; GM: geometric mean; GSD: geometric standard deviation; ppm: parts per million.

## 4 Discussion

To our knowledge, this is the first effort to explicitly analyze the OSHA CEH and IMIS/OIS databases for EtO, which include over 40 years of air monitoring data covering several industries and job categories. We found that exposures in industrial sterilization settings were significantly higher than exposures in health service and chemical manufacturing settings (see Table 1). We also observed reductions in EtO concentrations in workplace settings over time, but these reductions were not evenly distributed across key industries.

### 4.1 Industry and Occupation Findings

Industrial sterilization workers, irrespective of SEGs, exhibited the highest median exposure concentration (0.4061 ppm) and the highest likelihood of OSHA PEL and NIOSH REL exceedance in comparison to other SEGs.

Exposure differences across the industrial sterilization and health service SEGs were likely mediated by measurable differences in EtO usage volume per sterilization cycle between the two industries. Still, across all three industries, it was unclear whether exposures were captured during routine work activities or during nonroutine activities such as startup operations, shutdown operations, or unintended events such as accidental releases. Several studies have characterized exposure to EtO following nonroutine exposure events in chemical manufacturing settings by measuring EtO‐mediated adduct levels in worker's blood, but none have measured EtO concentrations in the breathing zone of workers during startup and shutdown operations or during unintended events [38, 39].

Across the published literature, differences in exposure by occupation were evident for both the health services and industrial sterilization industries. Within the health services industry, sterilization operators exhibited substantially higher exposures than other occupational categories [9]. Within industrial sterilization settings, sterilization operators consistently exhibited the highest exposure concentrations, followed by other occupations such as packers, stockroom workers, and maintenance workers [40, 41, 42]. However, because all occupation‐level data reported in the literature were captured before the 2000s and primarily outside of the United States, it is unclear how generalizable these literature findings are for current US workers with potential EtO exposure. Future studies may consider characterizing exposures for different occupations during both routine and nonroutine job activities to assess routine and peak exposures.

### 4.2 Temporal Findings

Differences in the patterns of exposure over time may partially be attributed to when key exposure mitigation strategies were implemented for each industry. For the chemical manufacturing sector, the most impactful exposure reduction strategy was likely the phasing out of the “dirtier” chlorohydrin process and the adoption of the “cleaner” direct oxidation process. This process change largely occurred in the 1960s and 1970s, before when OSHA investigators began collecting and reporting occupational air sampling results [30]. Since 2003, the EPA has implemented several standards to reduce hazardous air pollutants that include EtO environmental emissions at chemical manufacturing sites (e.g., National Emission Standards for Hazardous Air Pollutants) [43]. However, the general lack of workplace exposure data published since 2000 within the OSHA datasets (17%) and published literature makes it difficult to determine whether measurable reductions have truly occurred over time. The decrease in available EtO air monitoring data captured by OSHA investigators from the 1980s and 1990s to the 2000s was likely mediated by several factors. These factors include a shift in enforcement strategy from focusing on high‐risk industries to specific worksites with high injury rates, which has led to a reduction in the number of visits involving chemical exposure monitoring [44] and a reduced emphasis on regulatory enforcement of EtO exposures in workplace settings resulting from observable reductions captured in the 1990s, most notably in health service settings [45]. The low confidence in exposure reductions over time is illustrated by the widening error bars in Figure 3, particularly for the health services industry.

For the industrial sterilization industry, our analyses suggest that exposures peaked in the mid‐2000s, over two decades after the promulgation of the OSHA PEL and more than a decade following the establishment of the NIOSH REL. Increased implementation of exposure reduction strategies, such as increasing the number of purges per sterilization cycle and improved ventilation in sterilization and non‐sterilization areas (e.g., warehouse) likely contributed toward reductions in EtO exposure concentrations in the 2000s [46]. However, within the analyzed dataset, these reductions were not maintained, as exposures increased in the mid‐2010s. Given the small sample size representing industrial sterilization between 2010 and 2020 (*n* = 68), random variation likely played an important role in the sudden increase in EtO concentrations in the mid‐2010s; however, without adequate air sampling data during this period, it is difficult to surmise whether other factors played a role as well. More air sampling at industrial sterilization facilities would be helpful in determining whether exposures have generally increased at industrial sterilization facilities since the mid‐2010s or not.

For the health services industry, a consistent decrease in measured EtO exposure was observed from 1979 to 2020. The exclusive usage of combined sterilization‐aeration chambers (mandated by EPA in 2010) [47], improved ventilation in sterilizer rooms, and increased post‐sterilization purge cycles have likely contributed toward reductions in exposure over time [48, 49]. Steady exposure reductions in healthcare settings captured within the OSHA data during the 1980s and 1990s were corroborated by an independent study that evaluated air monitoring data in US hospitals between 1984 and 2001 [45]. However, few EtO air sample results associated with health service settings have been published by OSHA investigators or independent researchers since the early 2000s, suggesting the importance of increased air monitoring efforts in health service settings to determine more definitively whether exposure reductions have continued.

### 4.3 Limitations

One limitation of this study is that the samples represented in the OSHA datasets were not collected at random worksites. Historically, OSHA's site selection process for conducting compliance monitoring has focused on facilities with previous violations, worker complaints (e.g., samples exceeded OSHA PEL), or referrals of hazards from government or nongovernmental entities [50]. Several sites within the compliance datasets were sampled at two or more time intervals, presumably as follow‐up compliance investigations; this was also the case for the noncompliance dataset (state consultation dataset), but to a lesser degree (< 8% of samples). As expected, the compliance datasets, which accounted for 72% of the aggregated dataset, were associated with elevated exposure (median) and OEL exceedance compared to the state consultation dataset. However, given the lack of published industry EtO air monitoring data, it is unclear how the compliance and consultation data represented within the analyzed OSHA dataset compare to “baseline” levels for relevant industries and occupations. Additionally, of the samples associated with a reported sample duration (CEH dataset only), less than 10% were collected for 8 h or longer (median sample duration: 271 min), which could serve to reduce utility in a comparison with existing OELs given that current OELs are based on 8 or 10‐h TWAs. Still, of samples collected for 8 h or longer, the median exposure concentration and OSHA PEL and NIOSH REL exceedance fractions were marginally below that of samples collected for less than 8 h.

At the occupation level, geometric standard deviations were elevated for several SEGs, including the chemical manufacturing, animal service, and health service central processing workers (> 100). High geometric standard deviations could have been mediated by SEG misclassification, over‐broadened SEGs, or workplace‐to‐workplace differences in exposure magnitude. Still, the SEG findings largely reflect what has been reported in the literature, and geometric standard deviations for the industrial sterilization SEGs were much lower, indicating a better fit for these workers. Ultimately, sample results within the OSHA datasets may be more representative of “worst case scenario” workplace settings than what is typical at worksites handling EtO routinely. Nevertheless, understanding the upper boundary of exposure across different workplace settings that handle EtO is valuable, especially when conceiving relevant exposure risk models and determining policies and risk mitigation strategies for reducing exposures.

### 4.4 Current Occupational EtO Exposure Limits and Future Opportunities

Over the last 40 years, advancements in engineering control technologies have made it more feasible to maintain EtO exposures below current US OELs. Method sensitivity has also improved by multiple orders of magnitude, which has made it easier to capture EtO levels at and below the OELs, irrespective of sample duration (LOD of current OSHA method is 1.5 ppb, which is 67 times lower than NIOSH REL of 0.1 ppm) [51]. Workplace risk management strategies have also improved, and more stringent environmental regulations have been promulgated to mitigate EtO exposures in workplace settings, most notably within industrial sterilization settings where exposures have historically been the highest. Improved workplace management strategies and the increased implementation of controls have contributed to measured reductions in EtO levels across all three industries analyzed within the OSHA dataset. Since 2024, the EPA has amended the National Emission Standards for Hazardous Air Pollutants and the Federal Insecticide, Fungicide, and Rodenticide Act (FIFRA) (interim) to establish new requirements designed to reduce EtO exposures in health services and industrial sterilization workers, including but not limited to (1) stricter emission standards for sterilization room vents, (2) a ceiling limit for the concentration of EtO that can be introduced during a sterilization cycle (600 mg/L), and (3) and requirements for respiratory protections for workers at specific exposure thresholds [52, 53]. Under FIFRA, the EPA has also set a timeline for the establishment of an OEL for EtO in industrial sterilization settings that will first take effect in 2028 [54]. This OEL will start at 0.5 ppm before gradually decreasing over time to 0.1 ppm by 2035 and will be used to determine whether a worker must wear respiratory protection or not [54].

Epidemiological research published over the last 40 years provides additional supporting evidence that EtO is a human carcinogen [1]. The NIOSH cohort of over 18,000 EtO‐exposed workers provides some of the strongest evidence that EtO causes breast and hematopoietic cancer [1, 24, 55, 56]. In the most recent follow‐up of the NIOSH cohort, investigators estimated that women occupationally exposed to EtO for a decade at a level approximating the OSHA PEL died from breast cancer at a relative rate of 3.15 compared to unexposed women [57]. Similarly, investigators estimated that women occupationally exposed to EtO for a decade at a level approximating the NIOSH REL died from breast cancer at a relative rate of 2.28 compared to unexposed women [57]. Despite measured reductions in EtO levels across the health services, industrial sterilization, and chemical manufacturing industries between 1980 and 2020, our analysis of the OSHA dataset indicate that exceedance may still occur, particularly within the industrial sterilization industry for which NIOSH REL exceedance remained above 50% for EtO data collected between 2011 and 2020 (all industries: 12% exceedance fraction for OSHA PEL and 42% exceedance fraction for 2011–2020). Given the continued exceedance of existing US OELs and the growing epidemiological evidence of dose‐dependent carcinogenicity at occupationally‐relevant exposure levels, ample attention and efforts to ameliorate exposures to EtO across the health services, industrial sterilization, and chemical manufacturing industries are worthy of consideration.

### 4.5 Conclusion

Due to limited air monitoring data, the characterization of more recent exposure levels experienced by workers in the health services, industrial sterilization, and chemical manufacturing industries is insufficient. Still, despite the limitations of the aggregated OSHA dataset, our analysis indicates that a large proportion of exposures are occurring above the established exposure limits. This highlights the importance of stronger implementation of controls for the reduction of EtO across the three major industries represented, with a particular focus on the industrial sterilization industry. Increased exposure monitoring across key EtO‐exposed industries will help occupational health practitioners better characterize existing cancer risks to the US workforce and provide better data for practical public health interventions.

## Author Contributions

Brian Christensen, Kaitlin Kelly‐Reif, and Matthew Dahm established the framework for the paper. Brian Christensen and I‐Chen Chen conducted the statistical analyses and produced the figures and tables. Brian Christensen wrote the first draft, but all authors assisted in revising the manuscript. Brian Christensen addressed reviewer comments.

## Disclosure

The findings and conclusions in this report are those of the author(s) and do not necessarily represent the official position of the National Institute for Occupational Safety and Health and the Centers for Disease Control and Prevention.

## Ethics Statement

This activity was reviewed by the Centers for Disease Control and Prevention (CDC), deemed not research, and was conducted in accordance with applicable federal law and CDC policy.

## Conflicts of Interest

The authors declare no conflicts of interest.

## Supporting information

Supporting Information Document Clean.

## Endnotes

1 See 45 C.F.R. part 46, 21 C.F.R. part 56; 42 U.S.C. §241(d); 5 U.S.C. §552a; 44 U.S.C. §3501 et seq.

2 See, e.g., 45 C.F.R. part 46, 21 C.F.R. part 56; 42 U.S.C. §241(d); 5 U.S.C. §552a; 44 U.S.C. §3501 et seq.
