# Supplementary material for: Ethylene Oxide Measurements From OSHA Workplace Investigations: Patterns in Exposure by Industry, Occupation, and Over Time
Source: Am J Ind Med. 2025 Jul 21;68(10):845–55. doi: 10.1002/ajim.70008 (PMC12421703; doi:10.1002/ajim.70008)
Supplement: Supplementary file 1 — Supporting Information Document Clean. [file AJIM-68-845-s001.docx]

**Supporting Information**

Table S1. Regulatory and non-regulatory occupational exposure limits for ethylene oxide in air

| **Country** | **OEL Description** | **Duration** | **OEL Value (ppm)** |
| --- | --- | --- | --- |
| US (OSHA) | Regulatory | 8-hr | 1 |
| US (OSHA) | Short-term Regulatory | 10-minute | 5 |
| US (NIOSH) | Non-regulatory | 8-hr | 0.1 |
| US (NIOSH) | Short-term Regulatory | 10-minute | 5 |
| US (ACGIH) | Non-regulatory | 8-hr | 1 |
| Australia | Regulatory | 8-hr | 1 |
| Canada | Regulatory | 8-hr | 1 |
| Canada (Quebec Only) | Short-term Regulatory | short-term | 10 |
| Denmark* | Short-term Regulatory | short-term | 2 |
| EU | Regulatory | 8-hr | 1 |
| Germany | Non-regulatory | 8-hr | 0.1 |
| Germany* | Short-term Regulatory | 15-minute | 2 |
| Israel | Regulatory | 8-hr | 1 |
| Japan | Regulatory | 8-hr | 1 |
| Latvia* | Regulatory | 8-hr | 0.55 |
| New Zealand | Regulatory | 8-hr | 0.1 |
| China | Regulatory | 8-hr | 1 |
| Poland | Regulatory | 8-hr | 0.55 |
| Singapore | Regulatory | 8-hr | 1 |
| South Africa | Regulatory for GI | 8-hr | 2 |
| South Africa | Regulatory for Mining | 8-hr | 1 |
| South Korea | Regulatory | 8-hr | 1 |
| Sweden* | Short-term Regulatory | 15-minute | 5 |
| Netherlands* | Regulatory | 8-hr | 0.47 |

*Adopted more stringent occupational limits than European Union (EU) limit; EU countries all fall under EU regulatory limit of 1 ppm; GI: general industry; ppm: parts per million

Table S2a. Standard Industry Classification (SIC) Categorization Strategy for Health Services Industry

| SIC Value | Description of SIC | # of Samples | Justification |
| --- | --- | --- | --- |
| 741 | Vet services for livestock | 5 | Clear and obvious designation |
| 742 | Vet services for animal specialties | 42 | Clear and obvious designation |
| 8011 | Offices and Clinics of Doctors of Medicine | 36 | Clear and obvious designation |
| 8021 | Offices and Clinics of Dentists | 1 | Clear and obvious designation |
| 8043 | Offices and Clinics of Podiatrists | 1 | Clear and obvious designation |
| 8053 | Skilled nursing care facilities | 5 | Clear and obvious designation |
| 8060 | Hospitals | 4 | Clear and obvious designation |
| 8062 | General Medical and Surgical Hospitals | 1049 | Clear and obvious designation |
| 8063 | Psychiatric Hospitals | 4 | Clear and obvious designation |
| 8069 | Specialty Hospitals, except Psychiatric | 61 | Clear and obvious designation |
| 8071 | Medical Laboratories | 4 | Clear and obvious designation |
| 8072 | Dental Laboratories | 2 | Clear and obvious designation |
| 8093 | Specialty Outpatient Facilities, not elsewhere classified | 2 | Clear and obvious designation |
| 8099 | Health and Allied Services, not elsewhere classified | 34 | Clear and obvious designation |
| 8221 | Colleges, universities, and professional schools | 3 | Clear and obvious designation |
| 9199 | General government | 3 | Categorized under Health Services based on reported job title information. |
| 9233 | Correctional institutions | 2 | Categorized under Health Services based on reported job title information. |
| 9311 | Public finance, taxation, and monetary policy | 2 | Categorized under Health Services based on reported job title information. |
| 9451 | Administration of Veteran's Affairs, except Health & Insurance | 2 | Establishment (name) was used to determine that all 9451 datapoints belonged in the Health Services category. |
| 9531 | Administration of Housing Programs | 1 | Categorized under Health Services based on reported job title information. |
| 9711 | National Security | 11 | Contextual information such as the Establishment (name) and Job Title were used to delineate between data that was collected in health services settings and data that was not. |
| 9999 | Unclassifiable establishments | 5 | Categorized under Health Services based on reported job title information. |

Table 2b. Standard Industry Classification (SIC) Categorization Strategy for Chemical Manufacturing Industry

| SIC Value | Description of SIC | # of Samples | Justification |
| --- | --- | --- | --- |
| 2082 | Malt beverages (manufacturing) | 2 | Selected chemical manufacturing designation based on Establishment information. |
| 2673 | Plastics, foil, and coated paper bags | 3 | Selected chemical manufacturing designation based on job title information. |
| 2812 | Alkalies and chlorine | 8 | Clear and obvious designation |
| 2813 | Industrial gases | 6 | Clear and obvious designation |
| 2819 | Industrial Inorganic chemicals | 8 | Clear and obvious designation |
| 2821 | Plastics materials, synthetic resins, and nonvulcanizable elastomers | 9 | Clear and obvious designation |
| 2833 | Medicinal chemicals and botanical products | 3 | Samples represented were determined to likely be associated with chemical manufacturing activities based on job title information. |
| 2834 | Pharmaceutical preparations (manufacturing) | 1 | Lone datapoint representing 2834 that was placed into chemical manufacturing category was placed there based on job title information. |
| 2841 | Soap & other detergents, except specialty cleaners | 11 | Clear and obvious designation |
| 2842 | Specialty cleaning, polishing, and sanitation preparations | 7 | Contextual information such as the Establishment (name) and Job Title were used to justify categorization of samples into chemical manufacturing category. |
| 2843 | Surface active agents, finishing agents, sulfonated oils, and assistants | 40 | Clear and obvious designation |
| 2844 | Perfumes, cosmetics, and other toilet preparations | 7 | Clear and obvious designation |
| 2851 | Paints, varnishes, lacquers, enamels, and allied products | 6 | Clear and obvious designation |
| 2861 | Gum and wood chemicals | 2 | Clear and obvious designation |
| 2865 | Cyclic organic crudes and intermediates and organic dyes and pigments | 2 | Clear and obvious designation |
| 2869 | Industrial organic chemicals, not elsewhere classified | 39 | Clear and obvious designation |
| 2879 | Pesticides and agricultural chemicals | 6 | Clear and obvious designation |
| 2891 | Adhesives and sealants | 4 | Clear and obvious designation |
| 2892 | Explosives | 1 | Clear and obvious designation |
| 2899 | Chemicals and chemical preparations | 1 | Clear and obvious designation |
| 2911 | Petroleum refining | 2 | Clear and obvious designation |
| 3011 | Tires and inner tubes | 1 | Clear and obvious designation |
| 3069 | Fabricated rubber products, not elsewhere classified | 12 | Contextual information such as the Establishment (name) and Job Title were used to delineate between data that was collected in chemical manufacturing settings and data that was not. |
| 3081 | Unsupported plastics film and sheet | 1 | Clear and obvious designation |
| 3086 | Plastics foam products | 3 | Clear and obvious designation |
| 3089 | Plastics products, not elsewhere classified | 2 | Establishment (name) was used to determine whether companies should be placed into the "Chemical Manufacturing" category or the "Industrial Sterilization" category. |
| 3111 | Leather tanning and finishing | 2 | Selected chemical manufacturing designation based on job title information. |
| 3312 | Steel works, blast furnaces | 1 | Clear and obvious designation |
| 3354 | Aluminum extruded products | 2 | Selected chemical manufacturing designation based on job title information. |
| 5169 | Chemical and Allied Products | 14 | Clear and obvious designation |
| 7389 | Miscellaneous business services | 22 | Samples designated under chemical manufacturing were determined based on job title information. |

Table 2c. Standard Industry Classification (SIC) Categorization Strategy for Industrial Sterilization Industry

| SIC Value | Description of SIC | # Samples | Justification |
| --- | --- | --- | --- |
| 1731 | Electrical work (Electrical work except burglar and fire alarm installation) | 16 | Selected industrial sterilization designation based on job title information. |
| 2026 | Fluid milk | 1 | Clear and obvious designation |
| 2099 | Food preparations, not elsewhere classified | 82 | Clear and obvious designation |
| 2833 | Medicinal chemicals and botanical products | 1 | Lone establishment determined to be associated with spice sterilization activities. |
| 2834 | Pharmaceutical preparations | 21 | All Establishments with exception of 1 associated with medical equipment sterilization. |
| 3069 | Fabricated rubber products, not elsewhere classified | 11 | Samples placed into Industrial Sterilization category determined to partake in medical instrument sterilization activities based on Establishment (name) and job title information. |
| 3089 | Plastics products, not elsewhere classified | 62 | All represented establishments associated with production of hospital disposable equipment. |
| 3441 | Fabricated structural metal | 3 | All establishments represented associated with production of surgical instruments. |
| 3841 | Surgical and Medical Instrument Manufacturing | 153 | Clear and obvious designation |
| 3842 | Orthopedic, prosthetic, and surgical appliances and supplies | 153 | Clear and obvious designation |
| 3911 | Jewelry, precious metal | 14 | Lone establishments associated with ear piercing equipment production which require sterilization. |
| 4215 | Courier services, except by air (Motor freight transportation and warehousing) | 1 | Lone establishment represented determined to likely be associated with sterilization activities. |
| 4222 | Refrigerated warehousing and storage (Motor freight transportation and warehousing) | 1 | Lone establishment represented determined to likely be associated with sterilization activities. |
| 4226 | Special warehousing and storage, not elsewhere classified (Motor freight transportation and warehousing) | 4 | Establishments represented determined to likely be associated with sterilization activities. |
| 4783 | Packing and crating | 2 | Establishments represented determined to likely be associated with warehousing activities at sterilization facilities. |
| 5047 | Medical, dental, & hospital equipment and supplies | 23 | Clear and obvious designation |
| 5049 | Professional equipment and supplies, not elsewhere classified | 29 | All establishments represented associated with medical instrument manufacturing or medical equipment sterilization activities. |
| 5141 | Groceries, General Line | 2 | All establishments represented determined to likely be associated with food sterilization activities (likely spices). |
| 6324 | Hospital and Medical Service Plans | 3 | Lone establishment determined to be a contract sterilization company. |
| 7342 | Disinfecting and pest control services (services to dwellings and other buildings) | 3 | Clear and obvious designation |
| 7389 | Miscellaneous business services | 37 | All establishments represented associated with microbiology research (requires sterilization) or sterilization activities. |
| 8731 | Commercial, Physical, and Biological Research (Eng, accounting, research, management, and related services) | 6 | All establishments represented associated with microbiology research (requires sterilization) or sterilization activities. |
| 8734 | Testing laboratories (Eng, accounting, research, management, and related services) | 6 | All establishments represented associated with microbiology research (requires sterilization) or sterilization activities. |
| 8748 | Business consulting services | 2 | Establishments categorized under industrial sterilization based on establishment and job title information. |
| 8999 | Services, not elsewhere classified (Miscellaneous) | 29 | All establishments represented associated with microbiology research (requires sterilization) or sterilization activities. |

Table S3. Similar Exposure Group Criteria and Descriptions

| Similar Exposure Groups by Industry | Definition | Job Title Inclusion |
| --- | --- | --- |
| Chemical Manufacturing Worker | Workers operating at facilities where ethylene oxide is produced or used as an intermediate. | All chemical manufacturing job titles |
| Veterinarian Services | Individuals working at animal hospitals or clinics. | All veterinarian and veterinarian technician-related job titles |
| Central Processing Workers | Workers that perform sterilization-related job tasks including loading medical instruments into the EtO sterilization chamber, turning the sterilizer on, and unloading the sterilization chamber or workers that conduct maintenance on sterilization equipment. | Sterilize processing division technician, sterilize processing division attendant, technician, instrument (technician), medical supply technician, department aide, medical aide technician, central sterilize technician, supply preparation worker, material handler, decontamination work, central supply technician, maintenance, CPO tech, tissue bank technician, central processing supervisor, Senior technician assistant, SPP supply, SPD supply, sterilizer, SPC technician, SPDA, central services worker, chief SPD, CONTL supply lead, autoclave operator, supervisor medical supply, SPD manager, CSR technician, control service, control service supervisor, load technician, wrap and steam sterilizer, EtO operator, CSS loading, CCS unloading, process technician, CMS technician, supply technician, CSP technician, laborer during decontamination activities, CST |
| Other Healthcare workers | Health service workers that do not perform sterilization-related job activities or maintenance of sterilization equipment, but routinely handle medical instruments that have been sterilized. | Clerk, supervisor, assistant supervisor, operating room technician, department aide, laborer, surgical technician, respiratory therapist, packager, dental technician, registered nurse, orderly, nurse aide, attendant operating room specialist, operating room nurse, orderly operating room technician, custodian, secretary, pack technician, operator, LPN, air conditioning mechanic, supply and distribution, respiratory lab technician, worker, industrial hygienist certified safety and health official, surgical processing, distribution attendant, housekeeper, mechanic/engineering, CHS technician, delivery, director of surgery, service technician, certified safety and health professional, custodian |
| Industrial Sterilization Operator | Workers that perform sterilization-related job tasks including loading medical instruments into the EtO sterilization chamber, turning the sterilizer on, and unloading the sterilization chamber. | Gasser, Vacu-Gas Operator, aeration operator, sterilizer operator, technician, autoclave operator, sterilizer, sterilizer helper, sterility attendant, chemical process, material handler, lab technician, sterijet operator, operator, sterilization MGN, sterilization warehouse, Vacudyne operator, quarantine sample, EtO operator, non-sterile setup, outbound setup, lab testing, tank changing, EtO chamber operator, filler, field technician, quarantine |
| Industrial Sterilization Production Floor Worker | Supervisors, custodians, inspectors, maintenance workers, and other workers that are routinely present at the facility but are not involved directly in sterilization-related activities. | Window person, spice grinder, blending set-up man, retort operator, finishing serviceman, DCD clerk, machinist QC supervisor, laborer, machine operator, hot wire machine, OC manager, roto wrap operator, maintenance, custodian, MFG support technician, microbiologist, circle machine operator, street operator, group leader, camp machine operator, supervisor, quality assurance, floor supervisor, operator of mill, folder, inspector, production supervisor, replenisher, picker, assembly, prop test, crusher, press operator, routerer, mold operator, rotocast, R&D engineer, gluer, housekeeper, in-process inspector (IPI) |
| Industrial Sterilization Warehousing Worker | Workers that receive, store, and organize, package, and ship medical instruments (pre- and post-sterilization). | Receiving dock attendant, forklift operator, truck operator, packer, packaging clean side, warehouse worker, cutter/packer, E 70 operator, quarantine shipper, asst shipping, shipping and receiving, boxer, receiver, warehouse associate, warehouse, overwrapping operator, maintenance shipping, bar sealer operator, receiver, receiving coordinator, foreman, UPS room |

Figure S1. Flow Chart of inclusion and exclusion decisions stratified by dataset (top: Chemical Exposure Health (CEH) dataset; middle: state consultation dataset; bottom: Integrated Management Information System (IMIS)/OSHA Information System (OIS) dataset)

Table S4. Relevant OSHA Air Sampling Method Descriptions

| OSHA Method | Relevant Time period | Recommended Sample Duration (min) | Recommended Sample Volume (L) | LOD @ Recommended Sample Volume (ppb) | LOQ @ Recommended Sample Volume (ppb) |
| --- | --- | --- | --- | --- | --- |
| OSHA Method 30 | 1979-1984 | 20 | 1 | 13.3 ppb | 52.2 ppb |
| OSHA Method 50 | 1985-2006 | 240 | 24 | 3 ppb* | 3 ppb |
| OSHA Method 1010 | 2007-2020 | 240 | 12 | 0.44 ppb | 1.5 ppb |

*LOD of 1.4 ppb for analytical procedure, but overall procedure has LOD of 3 ppb, which is equivalent to LOQ for method.

Figure S2. Distribution of Aggregate and Individual Datasets


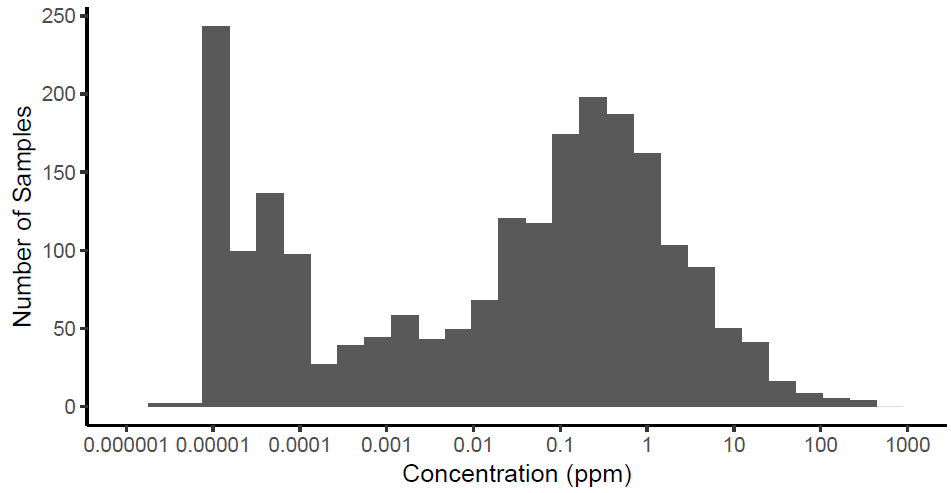


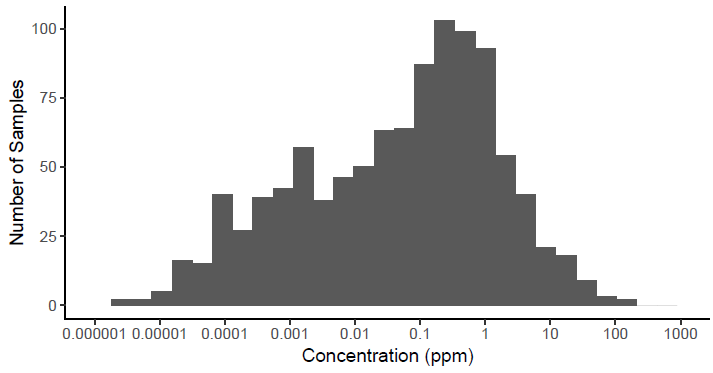


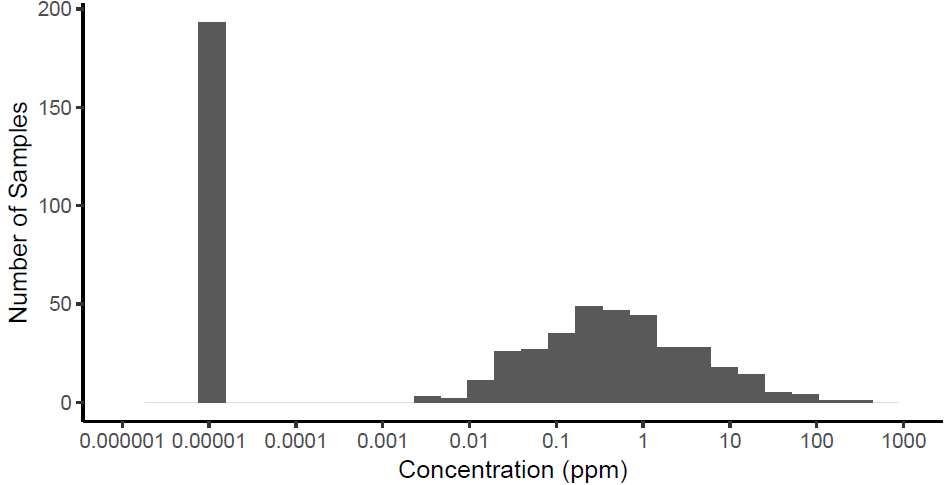


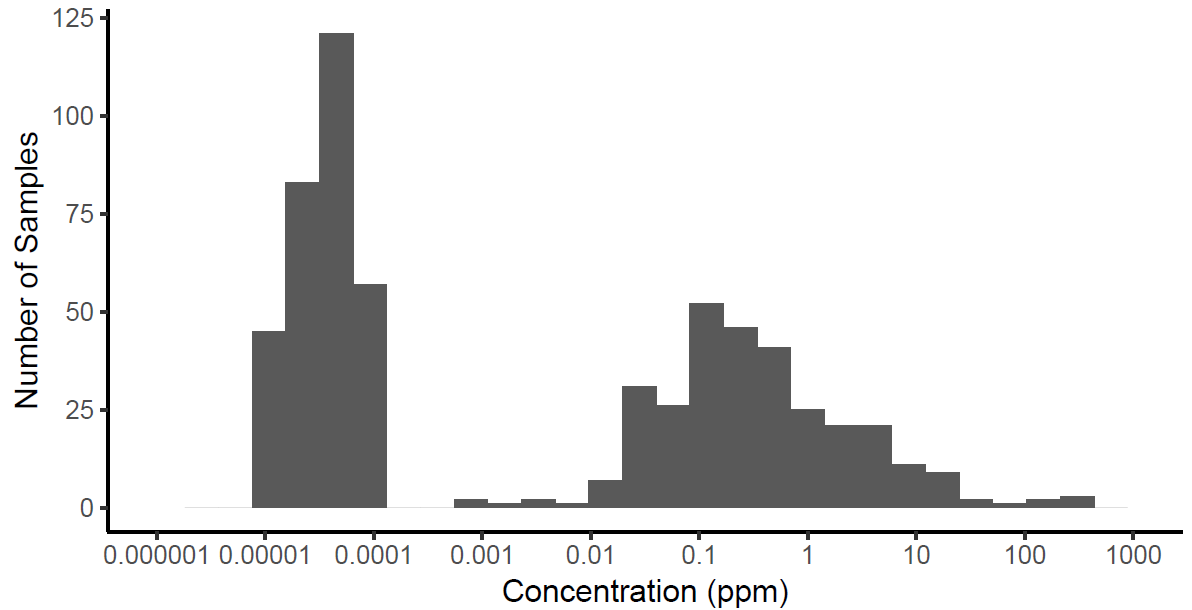


Aggregate Dataset (top), CEHD (2^nd^ from top), IMIS/OIS Dataset (3^rd^ from top), State Consultation Dataset (bottom); ppm: parts per million

Table S5. Airborne ethylene oxide concentrations stratified by several independent variables

| **Category** | **# of Measurements** | **DF%** | **Median (ppm)** | **GM (ppm)** | **GSD** | **%>PEL** | **%>REL** |
| --- | --- | --- | --- | --- | --- | --- | --- |
| Entire Dataset (1979-2020) | 2182 | 61% | 0.0600 | 0.0151 | 97.2 | 18% | 46% |
| CEH Dataset | 1035 | 66% | 0.0920 | 0.0437 | 33.3 | 19% | 49% |
| IMIS Dataset | 537 | 64% | 0.0890 | 0.0106 | 236.3 | 23% | 49% |
| State Consultation Dataset | 610 | 50% | 0.0001 | 0.0034 | 128.3 | 13% | 38% |
| Sample Type |  |  |  |  |  |  |  |
| Area | 436 | 65% | 0.0681 | 0.02053 | 96.0 | 20% | 65% |
| Personal Breathing Zone | 1721 | 60% | 0.0600 | 0.01433 | 97.0 | 18% | 46% |
| Citation Status |  |  |  |  |  |  |  |
| Citation given | 721 | 68% | 0.1000 | 0.02028 | 118.7 | 22% | 50% |
| No Citation | 869 | 52% | 0.0186 | 0.004821 | 121.7 | 12% | 38% |
| Type of Visit |  |  |  |  |  |  |  |
| Initial Visit | 543 | 47% | 0.0001 | 0.002565 | 130.7 | 13% | 34% |
| Follow-up Visit | 53 | 79% | 0.2220 | 0.04881 | 49.7 | 21% | 66% |
| Same Duration (minutes) |  |  |  |  |  |  |  |
| < 480 | 936 | 65% | 0.0950 | 0.0461 | 32.9 | 19% | 50% |
| ≥ 480 | 99 | 74% | 0.0764 | 0.0262 | 35.8 | 11% | 42% |
| < 271^*^ | 517 | 58% | 0.0586 | 0.0448 | 31.2 | 20% | 46% |
| ≥ 271^*^ | 518 | 74% | 0.1178 | 0.0426 | 35.6 | 18% | 52% |
| ≤ 60 | 267 | 52% | 0.0416 | 0.0568 | 31.4 | 23% | 45% |
| > 60 | 768 | 71% | 0.1000 | 0.0399 | 33.9 | 17% | 50% |
| ≤ 15 | 112 | 45% | 0.0316 | 0.0389 | 37.9 | 20% | 40% |

*Represents median sample duration for CEH dataset; DF%: detection frequency; ppm: parts per million; GM: geometric mean; GSD: geometric standard deviation; %>PEL: percent in exceedance of OSHA permissible exposure limit; %>REL: percent in exceedance of NIOSH recommended exposure limit
